# Evaluation of Real-Life Data on Toxicity in Elderly Patients Undergoing Adjuvant External Beam Radiotherapy for Endometrial Carcinoma

**DOI:** 10.3390/cancers18071061

**Published:** 2026-03-25

**Authors:** Kateryna Zarour, Robert Michael Hermann, Mirko Nitsche, Cedric Oliver Carl, Frank Bruns, Adrianna Cieslak, Daniela Meinecke, Mathias Alexander Sonnhoff

**Affiliations:** 1 Department of Radiotherapy, Hannover Medical School, Carl-Neuberg-Straße 1, 30625 Hannover, Germany; 2 Center for Radiotherapy and Radiooncology Bremen and Westerstede, An der Hössen 34, 26655 Westerstede, Germany; 3 Department of Radiation Oncology, University Medical Center Schleswig-Holstein, 24105 Kiel, Germany; 4 IMAGINE-Niedersachsen, Carl-Neuberg-Str. 1, 30625 Hannover, Germany

**Keywords:** radiotherapy in elderly, geriatric oncology, radiotoxicity, postoperative radiotherapy, endometrial carcinoma

## Abstract

Background/Objectives: Endometrial cancer is one of the most common malignancies of the female reproductive system. Fortunately, risk-adapted therapy offers a promising chance of recovery, even from locally advanced disease. In particular, older patients with higher risk factors benefit markedly from adjuvant radiotherapy with or without chemotherapy. Nevertheless, this also carries a risk of higher cumulative toxicity thereafter. Although this phenomenon has been observed in older patients, it has not yet been adequately evaluated. Methods: This retrospective study compared the clinical features of patients receiving adjuvant radiotherapy with or without sequential chemotherapy or radiotherapy with concomitant chemotherapy between 2011 and 2023. The cohort was divided into two groups: patients over 65 years old and those under 65. Results: 100 patients received adjuvant radiotherapy at our center between 2011 and 2023. The mean age of patients was 66.87 years. We observed that neither diabetes, obesity, concurrent, sequential chemotherapy, nor para-aortic field irradiation was associated with an increased incidence of acute radiogenic side effects such as cystitis, diarrhea, proctitis, radiodermatitis, nausea, or vaginal dryness according to CTCAE ≥ 2 (all *p* > 0.50). No difference was found between the two groups in terms of the incidence of radiotherapy-associated toxicity. Conclusions: We found no increase in toxicity in elderly patients after adjuvant radiotherapy with or without chemotherapy.

## Simple Summary

Endometrial cancer is one of the most common malignancies of the female reproductive system. Older patients with high-risk factors often benefit from adjuvant radiotherapy with or without sequential chemotherapy or concurrent radiochemotherapy, but these treatments can cause side effects. In a retrospective analysis of 100 patients treated between 2011 and 2023, the outcomes of patients aged over 65 years were compared with those of patients under 65 years. The study evaluated acute and long-term radiotherapy-related toxicities, including bladder inflammation, diarrhea, skin reactions, nausea, and vaginal dryness. Results showed no significant differences in toxicity rates between the two groups. Factors such as diabetes, obesity, sequential chemotherapy, and extended radiation fields were not associated with higher rates of moderate-to-severe side effects (CTCAE ≥ 2°). These findings indicate that adjuvant radiotherapy with or without chemotherapy does not lead to increased toxicity in elderly patients, and therefore may be considered as a safe option for appropriately selected individuals.

## 1. Introduction

Endometrial carcinoma is one of the most common tumors in women [1,2]. The prevalence of more-aggressive carcinomas is higher among older patients, who are less likely to participate in gynecological preventive measures, than younger patients [3,4]. However, even though locally advanced endometrial carcinomas are primarily operable, there are specific risk constellations where an adjuvant therapy is necessary to increase the chances of remission [5,6]. Further improvements in clinical endpoints can be achieved by using immunotherapeutic agents targeting the PD-L1 system, albeit in some cases only in subgroups [7,8]. This requires careful preselection, in order to weigh up the benefits against the expected toxicity of the therapy. Given the numerous options available for treatment regimens, selecting the option that offers the greatest benefit and the most acceptable risk in each situation, while weighing up the toxicity, is a challenge.

According to landmark trials, in most cases the advantages for oncological end-points outweigh the risk of toxicity from adjuvant radiotherapy with or without chemotherapy [9]. In addition to proving the efficacy of the therapy, identifying the subgroups that benefit and characterizing the endpoints, such trials also include an analysis of the expected toxicity. However, large studies often focus primarily on young patients, who only partially reflect epidemiological reality [10]. Although older patients are often included in such investigations, contributing to assessments of therapeutic effectiveness overall, simple conclusions cannot be drawn about toxicity in this particular age group. Lower tolerances for radiogenic toxicity among elderly patients are frequently discussed [11]. Due to an insufficient number of studies specifically examining elderly patients, there is no evidence on whether radiotherapy is less well tolerated among this age group. Herein, we compared the incidence of radiogenic toxicity in elderly patients with younger women who received standard clinical care. The aim of this study is to address this evidence gap in order to assess whether increased rates of toxicity are to be expected and, thus, to further specify the content of advice given to elderly patients regarding expected toxicity.

## 2. Materials and Methods

We conducted a retrospective study of patients with endometrial cancer who received an adjuvant radiotherapy with or without chemotherapy or concurrent radiochemotherapy with curative intent as part of their treatment. Data processing and evaluation adhered to the guidelines of the Ethics Committee of Hanover Medical School, Germany, 11165_BO_K2023. The Ethics Committee of Hannover Medical School agreed that there were no concerns regarding the study design that would prevent the survey from being conducted.

The study included patients older than 18 years, all of whom provided written consent for retrospective data processing following a curative treatment concept. Patients who received vaginal brachytherapy for endometrial cancer were excluded from this study as they were not treated at our center but referred to suitable facilities. Following treatment, these institutions took responsibility for further aftercare, meaning that we are unable to provide further data on these patients.

Demographic data, including age, height, and weight, were collected, as well as data on comorbidities and information on peri-oncological treatment. Irradiation relevant parameters were recorded, including the dose concept and the clinical course before, during, and after radiotherapy. The evaluation focused on both acute and chronic radiogenic side effects. These were assessed according to CTCAE 4.0 (Common Terminology Criteria for Adverse Events) and, subsequently, CTCAE 5.0. Acute side effects were obtained during radiation treatment as part of regular check-ups during therapy or immediately upon completion of radiation therapy. A significant difference between the CTAE 4.0 and CTAE 5.0 versions was examined during the study design phase; however, this could not be identified for the relevant toxicities described here. Late effects were assessed as part of structured follow-up care, consisting of an anamnesis and a physical examination, which is routinely offered at our center after three months and annually thereafter. If symptoms occurred, this assessment was carried out more frequently. Radiotherapy was administered as intensity-modulated therapy (IMRT). Indication was determined based on the valid version of the guidelines. Radiation therapy was performed in the pelvic area, including the pelvic lymphatic drainage pathways, following the German guideline which is analogous to the PORTEC trial [5,12,13]. If positive infra-renal para-aortic lymph nodes were detected during surgery, or if there was an imaging correlate, these were also included in the field.

The group was divided into two subgroups: patients older than 65 years of age [14] called elderly endometrial postop RT (E-PORT), and patients younger than 65 years (Y-PORT).

Descriptive statistics for the entire cohort were provided, along with an evaluation of the risk of side effects associated with the recorded perioncological parameters and comorbidities. The following is an assessment of the risk of radiotherapy side effects in the cohort of elderly patients compared to the cohort of younger patients. Due to the retrospective design of the study and the associated sample size, which could not be influenced, an a priori power analysis was not performed. A chi-squared test or a Fisher’s exact test was employed to analyze differences in categorical variables, and a Mann–Whitney U test or an unpaired *t*-test was used to investigate differences in continuous variables.

A *p*-value of <0.05 was considered significant. Statistical analysis was performed using Prism^®^ 10 for Mac (Version 8.00, GraphPad Software Inc., San Diego, CA, USA).

## 3. Results

**Figure 1:**
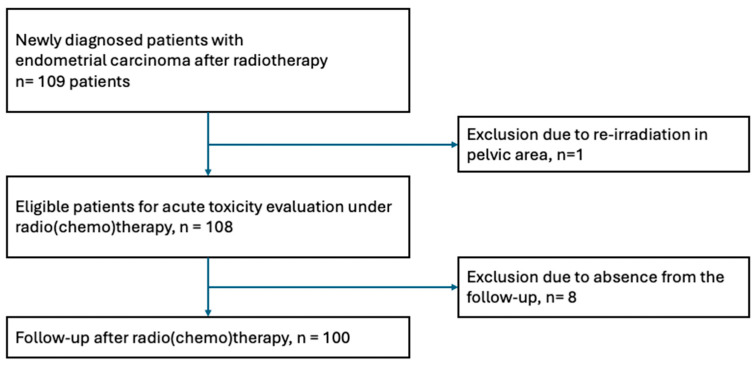
Flowchart.

Between 2011 and 2023, 109 patients were diagnosed with endometrial cancer and underwent radiotherapy with or without chemotherapy in the pelvic area. Only one patient was excluded from the study due to a re-irradiation situation in the pelvic region (Figure 1).

The mean age of the cohort was 66.9 years (SD = 10.2). Table 1 presents demographic data from 108 patients who received postoperative radiotherapy with or without chemotherapy, along with available toxicity data. A total of 100 patients with a follow-up no less than 3 months after receiving radiotherapy were included in the analysis. We observed that neither diabetes, obesity, concurrent chemotherapy, sequential chemotherapy nor para-aortic field irradiation were associated with an increased incidence of acute radiogenic side effects such as cystitis, diarrhea, proctitis, radiodermatitis, nausea, or vaginal dryness according to CTCAE >= 2° (all *p* > 0.50).

In the E-PORT group, the mean age in years was 73.3 (SD = ±5.91), compared with 56.4 (SD = ±6.23) in the Y-PORT group. In Y-PORT patients, radiation therapy of the para-aortic lymphatic drainage pathway was received significantly more often (*p* < 0.05) than in the E-PORT group. The treatment discontinuation rate was 5.97% in the E-PORT group and 10.25% in the Y-PORT group, a difference which was not statistically significant (*p* = 0.46) (details in Table 2). No higher incidence of CTCAE 2° toxicity was found in the E-PORT group (Table 3).

Late toxicity analysis was performed on 100 patients. Eight patients did not show up for the follow-up care. The median follow-up was up to 13 months after receiving radiotherapy.

A total of 24 cases of chronic cystitis were identified, all of which, except one, were CTCAE 1°. In the E-PORT group, there were sixteen cases of CTCAE 1° cystitis; in the Y-PORT group, there were seven cases of CTCAE 1° and one of CTCAE 2° cystitis. There was no significantly higher risk for the group (*p* > 0.5). Furthermore, no significant differences were documented in chronic proctitis: six per group with CTCAE 1° and only one with CTCAE 2° (in the Y-PORT group) (*p* > 0.5). During follow-up, at least three patients complained of vaginal dryness: one CTCAE 1° in each group, and at least one patient with chronic vaginal dryness CTCAE 2° (*p* > 0.5).

## 4. Discussion

Endometrial carcinoma is the sixth-most-common cancer in women [1], with increasing incidence [15] and a median age over 65 years [16,17]. This makes endometrial carcinoma patients a representative group in clinical routine as a common entity in the field of gynecological oncology. It is estimated that in the next few years the numbers of patients requiring treatment for endometrial cancer after the age of 65 will increase. Furthermore, patients older than 75 years constitute a large group of long-term cancer survivors [1]. However, the large studies that have defined treatment standards for locally advanced endometrial carcinomas [16,17] have recruited cohorts with a younger median age, e.g., GOG209 with a median age of 61 years, and PORTEC-3 with a median age of 62 years [18,19]. The over-65 age group was represented in these studies; however, the age-related risks of toxicity were not explicitly listed and examined. Even though the total study populations were larger than in our study, this issue was not dealt with extensively in these previous works. The postponement of recruitment, particularly of female patients, in these studies is evident in a comparison with our retrospective cohort from the clinical routine of adjuvant therapy for endometrial cancer. In our study, the median age is 67 years, which more accurately reflects the existing epidemiology of this malignancy in the European population. Therefore, it should also be asked whether age has a significant influence on the choice of treatment strategy or the scope of measures to be taken, without risking treatment that reduces quality of life. This particularly sharpens the advice that patients receive so that they can make a decision for themselves based on their individual preferences.

Furthermore, elderly patients are associated with more aggressive tumor characteristics which are independently but causally linked to poorer oncological outcomes [4,6,20]. However, we must also take into account that a radical approach may not always be warranted in the treatment of older patients [19]. This results in a need for therapy in high-risk constellations of locally advanced carcinoma, with a risk profile also in the group of elderly patients.

Regarding comorbidities such as diabetes or obesity, we have found no evidence that these significantly modulate or even negatively influence treatment tolerance with respect to therapy-related modalities such as radiation to the para-aortic lymphatic drainage pathway or the administration of chemotherapy. This also does not apply to the simultaneous application of chemotherapy, where toxicity increases, that is usually expected for other entities to improve oncological endpoints [21,22].

Furthermore, the introduction of IMRT, which provides better dose distributions in structures relevant to toxicity, also improves radiation therapy tolerance significantly [23,24]. For instance, IMRT reduces the rate of vaginal dryness up to more than 10% [25]. This is why an IMRT-associated technique has been a standard procedure at our center since 2010 for radiotherapy of malignant diseases in the pelvic region. The problem of vaginal dryness is more commonly known in the field of brachytherapy, but it can be controlled with appropriate accompanying treatment [26]. Similarly, such measures are also suitable for treating the rare complaints of vaginal dryness associated with IMRT.

Good tolerability of therapy is also reflected in the E-PORT cohort in our evaluation. The acute toxicity observed in the E-PORT group does not significantly differ from the acute toxicity observed in the Y-PORT group. The rates of treatment discontinuation are also equally low in both groups. Consequently, at least from the data of our routine cohort, taking into account the size and limitations of the study design, no higher toxicity can be assumed. The acute toxicity rates listed here for both groups are therefore comparable to those reported in the PORTEC-3 study for patients who received concurrent radiochemotherapy [27]. Our analysis did not reveal any instances of toxicity within our cohorts that exceeded the levels reported in prospective studies. This finding suggests that the treatment regimens administered to our patient group are associated with a similar or lower toxicity profile, compared to those documented in the existing literature from prospective clinical trials. Consequently, the concern that older patients might experience increased toxicity in real-world settings appears to be unsubstantiated, within the limits of our data.

In the context of clinical patient monitoring during ongoing therapy, any complaints outside the expected range would have been recorded and reported. It should also be added that part of the cohort comes from a pre-PORTEC 3 era. Nevertheless, we did not observe any toxicity that differed significantly in its severity from that reported in previous large studies, which tend to expand the scope of adjuvancy.

Furthermore, the majority of late toxicities were CTCAE 1°. This implies that the therapy is also well tolerated by elderly patients. Thus, even with the extension of the therapeutic standard, no significant increase in the risk of severe toxicity can be observed here. Therefore, it can be assumed that even in a group of older patients the treatment will be well tolerated, and that no limitations to the standard of care need to be introduced solely due to age or concomitant general diseases. The assumption that intensive therapy in this group reduces quality of life is a decision bias [28]. It has been shown that older patients also benefit from modern therapeutic approaches in the same way as younger patients [29,30]. The decision on treatment for endometrial cancer depends primarily on the individual patient risk profile [27,31,32]. This also applies to elderly patients, provided that the anticipated toxicity of postoperative radiotherapy is comparable. The toxicity appears to be low within the limits of this data set, and is comparable in both groups. It is imperative that patients receive comprehensive advice on the standard of treatment. Following this, a decision should be made on the scope of treatment in conjunction with the patient, taking into account the benefits, risks and costs of the treatment.

It is essential that an established treatment strategy is available for all patients, regardless of age. The decision-making process should not be influenced solely by the patient’s age, but should rather be guided by tumor characteristics and the patient’s individual preferences. This approach ensures that patients can receive adjuvant therapy according to standardized protocols without compromising quality of life. Maintaining access to standard treatment options among older patients allows for differentiated advice and supports shared decision-making, focusing on the needs and wishes of each patient.

The retrospective nature of this study is a major limitation. Due to the retrospective study design, no a priori power analysis was performed; therefore, potential underpowering of individual analyses cannot be ruled out. In particular, a single negative finding cannot be ruled out as a confounding factor, given the small number of cases and the monocentric data pool. Particularly for combination therapies and the various application regimens of chemotherapy, only limited conclusions can be drawn regarding the additive effects of toxicity. Furthermore, we did not include immunotherapeutic regimens in the present work, as these were not state-of-the-art at the time of patient treatment. Although this work offers clinicians comparisons with regard to decision making and clinical routine, it should be noted that patients are usually much older than those presented in the abovementioned studies. Structural follow-up care is necessary, in order to avoid missing relevant side effects in the therapy groups due to a lack of adherence to follow-up care [33]. The median follow-up of 13 months is unfortunately another weakness here, particularly with regard to long-term toxicity. This means, of course, that statements about late toxicity are only possible to a limited extent. The strength of the present study lies primarily in its description of acute toxicity during ongoing radiotherapy. This means that the statements presented here relate mainly to acute tolerability. Here, in particular, further expansion of individual patient counselling into subgroup-representative long-term follow-up studies of clinical routine is of considerable importance. More randomized studies should focus on this issue, to improve clinical standards in the future [34]. It is precisely here that prospective observational studies offer the greatest potential for making differentiated statements.

## 5. Conclusions

We did not identify any increased toxicity among older patients receiving curative radiotherapy with or without chemotherapy in this data set. Therefore, there is no indication to withhold curative therapy solely on the basis of age. Consequently, patients should receive adjuvant therapy in accordance with standard treatment based on tumor characteristics. This data can help in providing differentiated advice that increased toxicity due to age is not initially to be expected, and that there is still no clear indication of this. Thus, with adequate indication, consultation can continue to focus on the patient’s wishes in the sense of a shared decision. Further systematic, prospective studies or registries are warranted to elucidate the accurate estimates of real-world toxicity measurements in the future.

## Figures and Tables

**Table 1:** General patient characteristics. * No image-guided staging in the case of locally limited T1 findings.

Age (±SD) [y]	66.9 (±10.2)
Height (±SD) [m]	1.64 (±0.07)
Weight (±SD) [kg]	80.09 (±23.09)
BMI	29.67 (±7.95)
T1–1b [n]	55
T2–2b [n]	26
T3–4 [n]	27
N0 [n]	40
N1 [n]	39
N2 [n]	2
Nx * [n]	27
Diabetes (any type) [n]	17
Obesity [n]	29
Chemotherapy [n]	60
Concurrent	20
- Cisplatin weekly - Isofosfamid and Doxorubicin	17
	3
Sequential	40
- Carboplatin/Paclitaxel	
Para-aortic lymphatic drainage pathway	26
Mean PTV (±SD) [cm^3^]	1095 (±430.4)
Mean PTV Boost (±SD) [cm^3^]	305.2 (±391.0) *
50.4 Gy [n]	84
45 Gy [n]	14
Discontinuation of therapy	8

**Table 2:** Comparison between the elderly and younger-age patient groups.

	E-PORT *n* = 67	Y-PORT *n* = 39	Significance
Age (mean)	73.25	56.39	(*p* ≤ 0.05)
PTV [cm^3^]	1057	1158	(*p* = 0.27)
50.4 Gy [n]	54	30	(*p* > 0.50)
45 Gy [n]	9	5	(*p* > 0.50)
	4	4	(*p* = 0.46)
Pelvic lymphatic drainage pathway	53	25	(*p* = <0.05)
Para-aortic lymphatic drainage pathway	12	16	

**Table 3:** Comparison of acute and late toxicities between the E-PORT and Y-PORT groups.

	E-PORT	Y-PORT	Significant	E-PORT	Y-PORT	Significant
	Acute			Chronic		
Cystitis CTCAE 1°	28	17	(*p* > 0.50)	16	7	(*p* > 0.50)
Cystitis CTCAE ≥ 2°	9	5		0	1	
Diarrhea CTCAE 1°	17	23	(*p* > 0.50)	9	7	(*p* > 0.50)
Diarrhea CTCAE ≥ 2°	18	24		4	3	
Proctitis CTCAE 1°	8	16	(*p* > 0.50)	6	4	(*p* > 0.50)
Proctitis CTCAE ≥ 2°	4	7		0	1	
Dermatitis CTCAE 1°	6	9	(*p* > 0.50)	1	2	(*p* > 0.50)
Dermatitis CTCAE ≥ 2°	2	1		2	0	
Nausea CTCAE 1°	14	21	(*p* > 0.50)	6	4	(*p* > 0.50)
Nausea CTCAE ≥ 2°	4	4		0	1	
Vaginal Dryness CTCAE 1°	9	7	(*p* > 0.50)	1	1	(*p* > 0.50)
Vaginal Dryness CTCAE ≥ 2°	1	1		1	0	

## Data Availability

The data presented in this study are available on request from the corresponding author.

## Abbreviations

E-PORT	Elderly Endometrial Postoperative Radiotherapy
Y-PORT	Young Endometrial Postoperative Radiotherapy
CTCAE	Common Terminology Criteria for Adverse Events
IMRT	Intensity-Modulated Radiotherapy
